# Early Results from a Pressureless Middle Ear Diagnostic and Its Relation to the Types of Tympanometry Results

**DOI:** 10.3390/audiolres16030062

**Published:** 2026-04-22

**Authors:** Daniel Polterauer-Neuling, Maike Polterauer-Neuling, Peter Zoth, Carmen Molenda

**Affiliations:** 1 Section Cochlear Implantation, Department of Otorhinolaryngology, University Hospital of Munich (LMU), 81377 Munich, Germany; maike.neuling@med.uni-muenchen.de (M.P.-N.); carmen.molenda@med.uni-muenchen.de (C.M.); 2 Independent Freelance Scientist, 82205 Gilching, Germany; zothpeter1@zoth.eu

**Keywords:** hearing, auditory, tympanometry, infection, paracentesis, acoustic impedance, middle ear, ventilation tube

## Abstract

Background/Objectives: In addition to the clinical gold standard, tympanometry, several alternatives for middle ear diagnostics have evolved over the past decades. With the so-called pressureless acoustic impedance test, the Neuranix Medwave, another device, came into play. Methods: Using a retrospective, anonymous study design, descriptive data were reported, and the correlation between Medwave’s results and tympanometry types was evaluated. Also, the correlation between the patients’ age and the Medwave resulting parameters was evaluated. We were able to show changes in the measurement results over time in the case of paracentesis and tube insertion. Results: The analyzed data show that it is possible to differentiate between tympanometry result type A and type B using the Medwave resulting parameter resonance frequency (“fR”), but not when using peak admittance (“P”). Between all other types, it was not possible to differentiate using the Medwave resulting parameters, nor fR nor P. Due to the low statistical power, this may be due to a type II error. Regarding age, a correlation was found only for the tympanometry result type A. The case over time showed a clear difference in the affected ear between the time before and after the ear surgeries, as well as the contralateral healthy ear. Conclusions: While this study indicates the potential use of the PLAI technology, especially as a tool in situations where traditional tympanometry is not feasible, the results need to be interpreted with caution. Further validation with larger and more balanced groups of participants is necessary to confirm these initial findings and to more clearly define the clinical utility of this technology.

## 1. Introduction

Traditional methods investigating the function of the middle ear (e.g., otoscopy and tone audiometry) are highly subjective as they rely on visual cues and patients’ reports. As these patients’ reports are highly subjective and are memory-based, they are likely to be unreliable in many cases. For example, the reported or documented patient history cannot rule out a condition like otitis media, which may be asymptomatic [1].

Over the past century, objective middle ear diagnostics have evolved significantly. In the 1940s, tympanometry was developed, and it became widely used in the 1960s. Tympanometry measures the mobility of the tympanic membrane in response to changes in air pressure and, therewith, analyzes the middle ear function. In the 1970s, multi-frequency tympanometry enhanced diagnostic options without getting widely adopted in clinical applications [2].

All around the world, tympanometry has become a standard procedure in patients; although it is a non-optimal measurement. On the one hand, the accuracy in predicting middle ear effusions is poor. It can be incorrect in around 50% of the cases [3]. On the other hand, there is a need for air pressure applied to the outer ear canal, as it measures the mobility of the tympanic membrane in response to changes in air pressure. It therefore needs a sealed ear canal, which can be uncomfortable for patients and challenging for the audiometry staff to achieve, and, in some cases, even impossible (e.g., perforation of the tympanic eardrum, radical cavity, gaping tube, or myringosclerosis) [4,5,6,7,8].

Most recently, optical coherence tomography has been shown to allow a non-invasive imaging of the middle ear and provide real-time diagnostics in patients with otosclerosis or acute otitis media [9]. The positive aspect of optical coherence tomography is its high resolution. However, the negative aspect is a limited view and the need for special training for analyzing measurement results [10]. In addition, machine learning has been shown to improve diagnostic accuracy, especially in children, which enables the identification of middle ear effusions [11]. These innovative technologies are performed in addition to established techniques for a more precise and reliable diagnosis of the middle ear [12]. Besides these, many other approaches have been investigated in research; most of them remain experimental and are not, or at least not widely, used in clinical practice [13].

A potential improvement to traditional tympanometry is the so-called wideband tympanometry. In contrast to the single frequency used in traditional tympanometry, this technique uses a stimulation signal of a wide range of frequencies and analyzes the wideband absorbance. The wide range of analyzed frequencies aims to investigate more useful insights into the middle ear function (e.g., ossicular chain pathologies, superior semicircular canal dehiscence, or otosclerosis) [3,14,15].

In this article, we want to take a closer look at another new method for analyzing middle ear function similar to tympanometry. The applied pressure and needed sealing are known problems in tympanometry. Though some passive cooperation is needed [16], especially for neonates, they are difficult to test [17]. The main difference is that the Neuranix Medwave does not apply any pressure to the patient and is done in a few seconds. Therefore, it is not limited by issues like the mentioned discomfort of air pressure and the partly challenging sealing of the outer ear canal. But as it is a new technique, there is a need for clinical investigation regarding handling, ease of interpretation, and reliability of the results.

## 2. Materials and Methods

This observational retrospective, monocentric study included all patients from 2024 to 2025 who were tested using Medwave (Neuranix Srl, Gardigiano di Scorze, Italy) [18,19]. We used two Medwave devices with the software version 4.01.03.

To perform the measurement using the Medwave device, it is necessary to use soundproof earplugs to reduce ambient noise. Note that one benefit of this new measurement technique is that the device does not need pressure-tight earplugs, which are still crucial in standard tympanometry. Here, they help maintain consistent pressure in the ear, reducing discomfort induced by pressure changes during the test.

Before measurement, we selected individual standard earplugs for every patient. Sanibel ADI earplugs (Sanibel Supply, Middelfart, Denmark) were used in various forms (i.e., cap, flanged, and flanged with additional lamellae), ranging from 3 mm to 19 mm.

**Figure 1:**
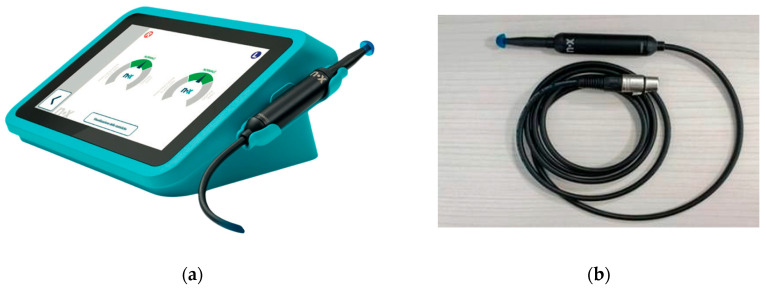
(**a**) Measurement device Medwave to perform the analysis of the functional status of the middle ear using the PLAI (Pressure Less Acoustic Immittance) method. (**b**) Probe of the Medwave^®^ device with an earplug. (The copyright of this figure belongs to Neuranix Srl, Gardigiano di Scorze, Italy).

The Medwave device (see Figure 1a) is an active diagnostic medical device for measuring middle ear impedance without air pressure variation in the ear canal. It is a device based on the so-called PLAI (Pressure Less Acoustic Immittance) technology, which assesses the functional status of the middle ear by quantifying the properties of the eardrum when exposed to a wide range of sound frequencies. The ability to perform the test without changing the pressure in the ear canal allows Medwave to be used pre-, intra-, and postoperatively to check the condition of the eardrum perforation.

The device consists of a calibrated impedance probe (see Figure 1b) which is coupled to the ear canal via an earplug. The probe primarily consists of a tube in which two microphones and a loudspeaker are positioned at a well-defined distance. The two microphones are positioned to pick up the speed of the moving air molecules to measure the velocity of the sound. The loudspeaker is located at the end of the tube. By measuring the sound velocity, the acoustic impedance/admittance can be easily determined (Impedance = Pressure (“p”)/Velocity (“v”)).

The probe is inserted into the external auditory canal. Care must be taken to ensure that the probe is not moved during the measurement and that it remains positioned longitudinally to the external auditory canal, in the same direction that would ideally allow observation of the eardrum. Compliance with this measurement condition is essential because probe movements or vibration could cause artifacts, and a wrongly chosen angle could generate impedance signals.

By inserting the Medwave probe into the external auditory canal, Medwave automatically initiates a calibration procedure and, afterward, the impedance measurement. The operating principle is based on state-of-the-art electronic components (precision microphones, MEMS micro–electro-mechanical systems) that are capable of measuring the acoustic signal in the ear canal and calculating the complex acoustic admittance (Y) based on the evaluation of the response in a range between 100 and 1500 Hz (defined by the manufacturer and covering the specific resonance frequencies known to be found in pathologic ears [20,21] and the standard frequencies used in tympanometry [22]). The applied chirp is in the range of 100 to 3000 Hz. The complex acoustic impedance (Z) is an indicator of how the applied acoustic energy is absorbed by the eardrum and the ear system in general and is a function of frequency. Admittance (Y) is defined as the inverse of impedance and is often used as an equivalent alternative. When the modulus of impedance is minimal, the admittance is at its maximum. The result of the measurement performed with Medwave is expressed in objective numerical terms:

- Ve = equivalent volume of the ear canal;
- fR = value of the frequency at which the maximum value (P) of the admittance curve (module) occurs;
- P = peak admittance value (module).

All shape parameters necessary for classification are obtained from the recorded curves.

**Figure 2:**
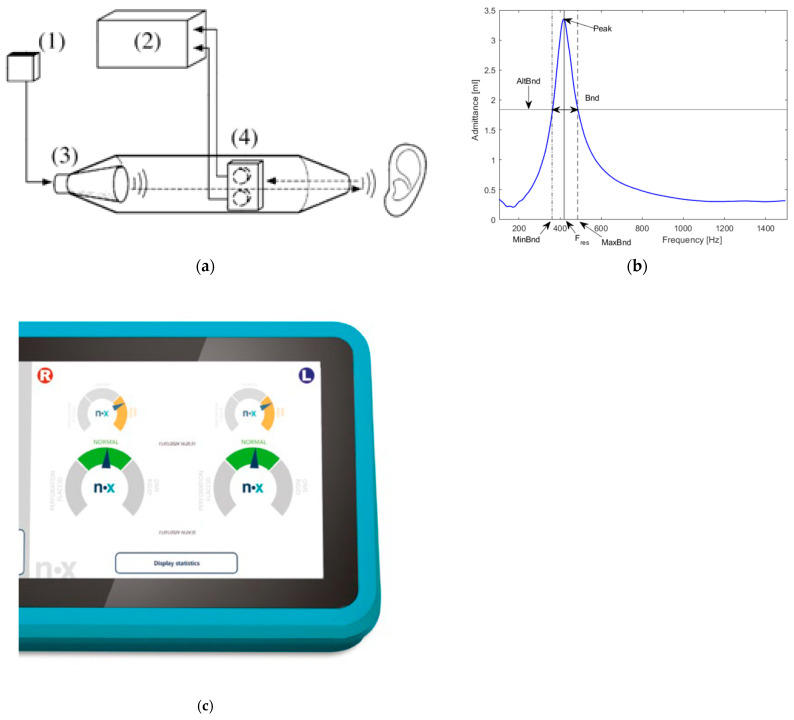
(**a**) Schematic of the Medwave device using Pressure Less Acoustic Immittance (PLAI), including its components: (1) sound source, (3) speaker, (4) microphones, and (2) processing unit for data analysis. (**b**) Example of a response curve from PLAI with parameters extracted from the curve, including the main parameter’s resonance frequency, here called F_res, and the curve’s peak [23,24]. (**c**) Example of a result of right and left ear shown on the medwave device screen. (The copyright of this figure belongs to Neuranix Srl, Gardigiano di Scorze, Italy.)

A technical scheme is shown in Figure 2a, while Figure 2 indicates an example of a response curve recorded by the Medwave device. In addition to the numeric frequency response, the device indicates a result: like normal eardrum = green, stiff eardrum = orange, and flaccid eardrum = orange.

As a comparison measurement, tympanometry was performed using the GSI 39 (gsi (Granson-Stadler), Eden Prairie, MN, USA) [25] or the eTymp (Merz Medizintechnik GmbH, Reutlingen, Germany) [26]. The resulting tympanometry curves were separated into the following types, shown in Table 1 [27,28,29]. In contrast to the standard procedure, we differentiated type C into two subtypes called “C−” and “C+” to pay attention to whether the peak was shifted to the left (negative pressure) or to the right (positive pressure). As recommended in the literature, we analyzed the mobility of the eardrum using a probe tone of 226 Hz as a standard value and 1000 Hz for patients below one year of age due to a lower ear canal volume and resulting higher resonance frequency [22,30,31].

In addition, for some patients, the otoscopy results or known normal hearing without any known health issues were included in the study’s retrospective analysis. For otoscopy, our ENT department uses the “HEINE BETA 200” diagnostic otoscope (HEINE Optotechnik GmbH & Co. KG, Gilching, Germany) [32] or Zeiss Opmi Pico surgery microscope (Carl Zeiss Meditec AG, Jena, Germany) [33].

The anonymous data export was performed using Innoforce ENTstatistics (Ruggell, Liechtenstein), analyzing all patients via the export tool. This concept was approved by our university ethics commission (project number 24-0923).

In addition to the retrospective analysis, we had a closer look at an individual case of paracentesis and, later, a second paracentesis, including tube insertion. In this case, we received individual consent from the patient to use her data in this manuscript.

To collect the relevant literature of this manuscript’s topic, besides manual search, we used ScienceOS (June 2025 version, https://www.scienceOS.ai, accessed on 17 June 2025). To support the translation of terms, we used DeepL Translate (June 2025 version, https://www.deepl.com/, accessed on 17 June 2025). To check grammar, we used Grammarly (June 2025 version, https://www.grammarly.com/, accessed on 17 June 2025).

The data analysis for this paper was generated using the Real Statistics Resource Pack software Release 9.3 (Copyright (2013–2025) Charles Zaiontz, www.real-statistics.com (accessed on 1 December 2024)). Numerical values were tested for normal distribution using the Shapiro–Wilk test. The two-tailed Mann–Whitney Test for Two Independent Samples was performed to test for significant differences. Holm’s method was used to correct the results of the Mann–Whitney Test for Two Independent Samples in multiple comparisons. Spearman’s rank test was performed to test correlations. Probability values of *p* < 0.05 were considered significant.

## 3. Results

### 3.1. Descriptive Analysis of the Retrospective Data

The retrospective analysis found 106 patients (102 patients bilaterally, 3 patients left-sided, and 1 right-sided) tested by the Medwave device in the time range of 2024 to 2025. This results in 208 ears tested by the Medwave device. The mean age of these patients was 33.48 +− 23.92 years with a minimum value of 0 years and a maximum value of 87 years. There were 48 male patients and 58 female patients. A total of 26 patients had no hearing problems bilaterally, and 13 had a single-sided hearing problem. This results in 65 ears with no hearing problems. The onset of hearing loss was documented in the following categories: no hearing loss, prenatal hearing loss, hearing loss during the 0–1-year age range, during the 1–5-year age range, during the 5–20-year age range, and hearing loss after the age of 20. The 65 ears with no hearing loss were matched between documentation of etiology and the start of hearing loss. A total of 36 ears had a start of hearing loss during the prenatal phase. No ear was categorized at the start of hearing loss between 0 and 1 years of age. A total of 26 ears had a start of hearing loss between 1 and 5 years of age, while 7 ears suffered from hearing loss between the ages of 5 and 20 years. In 48 ears, the hearing loss started after the age of 20.

In 106 ears, a type A tympanogram or no known ear problem was found (in case of result uncertainty, verified by otoscopy. As in one case, only one ear was measured in a bilateral type A case; only 105 ears were measured with the Medwave device. For further analysis, these ears were all stated as type A. In seven ears, a type As was found. All of them were measured with the Medwave device. In three ears, a type Ad was found. All of them were measured with the Medwave device. Type B was found in 19 ears. All but one ear of these were measured with the Medwave device. Type C+ was found in two ears (one measured by the Medwave device), while type C− was found in 13 ears (all of them measured by the Medwave device). In two ears, the tympanometry could not be performed due to sealing problems. In the remaining 60 ears, no corresponding tympanometry was available for comparison with the Medwave measurement.

**Figure 3:**
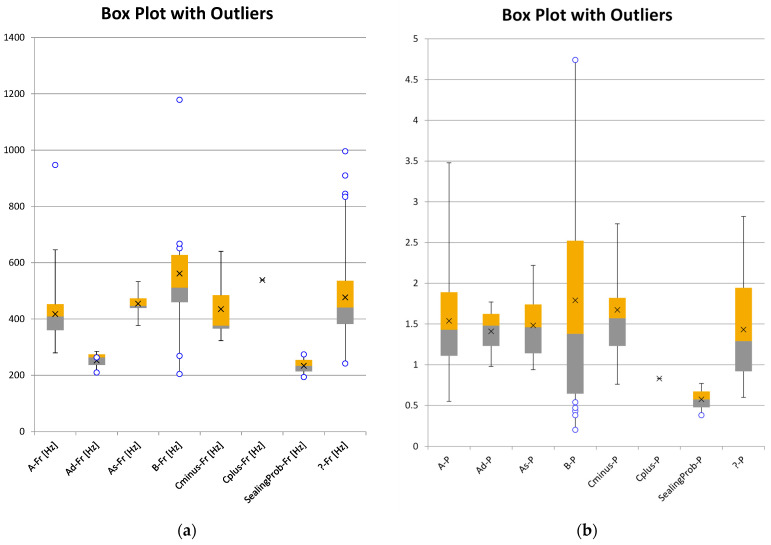
Boxplots with outliers of the resonance frequency fR (see sub-figure (**a**)) and the peak admittance P (see sub-figure (**b**)) given as a result parameter from the Medwave device’s impedance measurement, divided into tympanometry type sub-groups. Count for Fr as well as P: type A = 105; As = 7; Ad = 3; B = 19; C+ = 2; C− = 13.

The overall mean of all Medwave measurements with existing tympanogram showed a resonance frequency fR of 433.52 +− 132.14 Hz and a peak admittance P of 1.56 +− 0.73 10^−2^ mmho. In the next step, the Medwave results were separated into groups given by the tympanogram types (see Table 1). For type A, we found a mean fR of 417.85 +− 8.37 Hz and a P of 1.54 +− 0.06 10^−2^ mmho (n = 105). For type As, we found a mean fR of 454.50 +− 18.04 Hz and a P of 1.48 +− 0.17 10^−2^ mmho (n = 7). For type Ad, we found a mean fR of 253.01 +− 22.41 Hz and a P of 1.41 +− 0.23 10^−2^ mmho (n = 3). For type B, we found a mean fR of 561.96 +− 61.07 Hz and a P of 1.79 +− 0.33 10^−2^ mmho (n = 18). For type C+, we found a fR of 538.33 and a P of 0.83 (n = 1). For type C−, we found a fR of 435.22 +− 30.31 Hz and a P of 1.67 +− 0.19 10^−2^ mmho (n = 13). For the group with unknown corresponding tympanometry type, we found a mean fR of 476.55 +− 18.85 Hz and a P of 1.43 +− 0.08 10^−2^ mmho (n = 59). As a boxplot with outliers for each sub-group, the fR data is shown in Figure 3a, and the P data is shown in Figure 3b.

### 3.2. Group Comparisons of the Measured Medwave Parameters

As the groups Ad, C+, and sealing problem were of a small sample size, no comparison to other groups was possible. To compare the larger groups, the Shapiro–Wilk Test was used to evaluate the distribution for normality, as only one group had more than 50 samples.

For the parameter Fr, group As showed a normal distribution, while A, B, and C− were not normally distributed. Therefore, we performed statistical tests for non-normally distributed samples. Comparing group A, group As, group B, and group C−, only for group A vs. B was a significant difference found regarding the parameter fR (U = 497.5; n1 = 105; n2 = 18; *p* = 0.001; effect size r = 0.29; corrected due to multiple comparisons). All other comparisons of groups showed no significant difference regarding the parameter fR (*p* > 0.05).

For the parameter P, group A, group As, group B, and group C− were not normally distributed (*p* < 0.05). Comparing group A, group As, group B, and group C−, no significant difference was found for any group regarding the parameter P (*p* > 0.05).

### 3.3. Correlation of Patients’ Age and Measured Medwave Parameters per Group

In Section 3.2, we do not have a normally distributed dataset. Therefore, correlation testing was performed using Spearman’s rank test.

For the parameter fR, a correlation with the patient’s age was found for group A (ϱ (105) = −0.49; *p* < 0.001) and for group C− (ϱ (13) = −0.62; *p* = 0.02). No correlation for the parameter fR with patients’ age was found for group As (ϱ (7) = −0.04; *p* =0.94) and group B (ϱ (18) = −0.07; *p* = 0.77).

For the parameter P, a correlation with the patient’s age was found for group A (ϱ (105) = 0.20; *p* = 0.03). No correlation for the parameter P with patients’ age was found for group As (ϱ (7) = −0.26; *p* = 0.57), group B (ϱ (18) = 0.35; *p* = 0.36), and group C− (ϱ (13) =0.11; *p* = 0.73).

**Figure 4:**
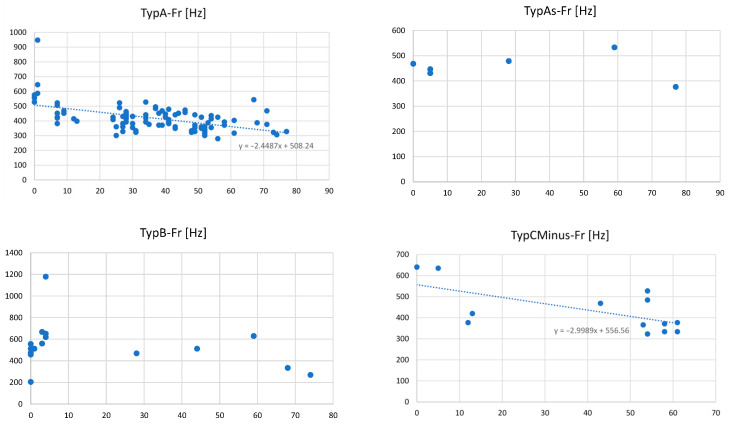
Correlation plots of value Fr [Hz] vs. patient’s age for tympanogram types A, As, B, and C−.

To visualize Fr vs. patient’s age, a correlation plot for the tympanogram types tested in this sub-section (see Figure 4). As no non-linear correlation could be inspected, no further analyses like regression have been applied.

### 3.4. Illustrative Case of Paracentesis and a Ventilation Tube in an Adult Subject

In a 44-year-old female patient, the initial subjective right-sided ear symptoms (pressure, pain, and glugging in the middle ear, as well as hearing loss) occurred in December 2024 and worsened till the beginning of 2025. To avoid pain in this subject, we chose Medwave’s PLAI test to analyze the current tympanic status.

**Figure 5:**
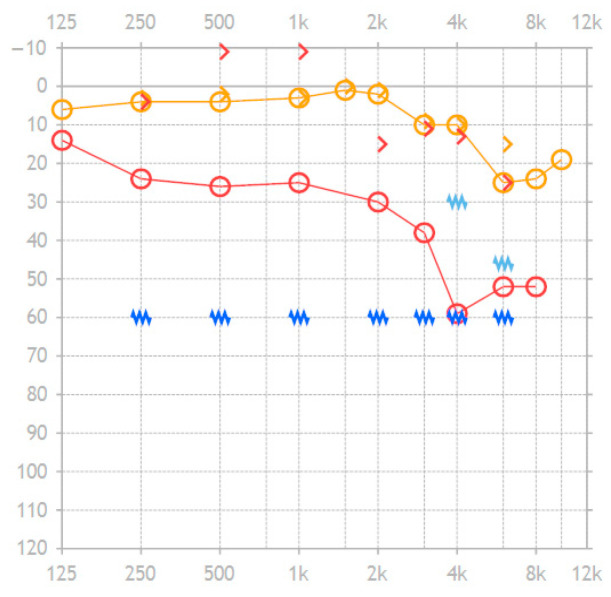
Tone audiogram of the reported individual case’s affected right ear before suffering from the reported symptoms (orange symbols) and during the symptoms but before the two surgeries (red symbols). The light blue and dark blue symbols show masking values for the orange resp. red curve. Circles are for air conduction, while arrows are for bone conduction. (The copyright of this figure belongs to INNOFORCE Est.).

On 8 January 2025, a tympanometry showed type B on the right side and type A on the non-affected left side. Therefore, a paracentesis was performed on the right side. In addition, a tone audiogram was performed showing an air-bone gap of 27.0 dB in the range of 500 Hz to 2 kHz and 31.8 dB for the range of 500 Hz to 4 kHz. The pure tone audiogram worsened by 24.0 dB in the range of 500 Hz to 2 kHz and by 30.3 dB in the range of 500 Hz to 4 kHz. The bone conduction showed an improvement of 1.5 dB. The tone audiogram data is shown in Figure 5.

On 10 January 2025, the first surgery took place. The ear inspection showed cerumen in the outer ear canal, which was removed, and the intact eardrum showed inflammation and was less opaque. The paracentesis was performed under local anesthesia in the anterior lower quadrant. The serotympanon was suctioned. The paracentesis did not solve the patient’s symptoms, and the tympanometry still showed type B on the right side and type A on the non-affected left side. Another surgery was performed. Therefore, after the re-paracentesis and suction of a serotympanon, a 1.25 mm titanium ear tube was inserted.

**Figure 6:**
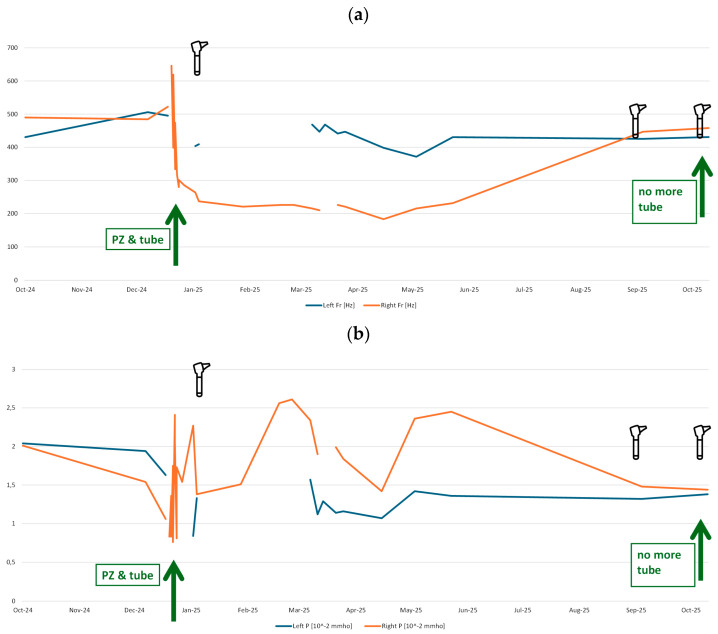
Medwave (Pressure Less Acoustic Immittance) test results over time showing the resulting values for (**a**) fR and (**b**) P for the right (orange) and left (blue) ear in the individual adult case of paracentesis and later on paracentesis plus tube insertion. The green arrows highlight the surgeries and the removal of the tube. In both sub-figures, the three otoscope icons (Otolaryngologist icons created by Rodrigo Guerios–Flaticon) mark the timepoints of the images shown in Figure 5.

Several ‘pressureless’ measurements could be performed using the Medwave device before, on the day of, and after the surgeries. Parallelly, tympanometry was performed regularly, showing type B for the affected right side. The measured values of fR and P can be found in Figure 6a and Figure 6b, respectively, showing the patient’s progression over time.

**Figure 7:**
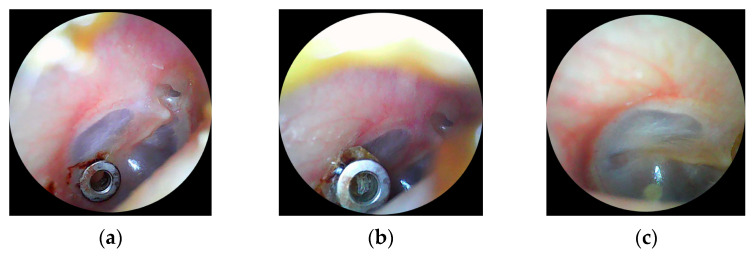
Otoscopic view of the eardrum and the tube at the stage of open tube and fR around 250 Hz (**a**). Otoscopic view of the eardrum and the tube at the stage of closing and fR around 500 Hz (**b**). Otoscopic view of the eardrum and the tube right after removing the tube (**c**).

The resonance frequency fR over time shown in Figure 6a indicates a clear difference between the right and left ear. On the right ear, it decreased significantly from approximately 500 Hz to around 250 Hz. This effect lasted for months (until 25 May) and rose back to the values of the contralateral ear in 25 September. Interestingly, the tube was not completely rejected from the tympanic eardrum at that time. The eardrum seemed closed on the images from the patient’s otoscopic camera integrated in a consumer ear cleaner (Bebird EarSight M9 S, app version 5.3.90, Heifeng Zhizao (Shenzhen) Technology Co., Ltd., Shenzhen, Guangdong, China). Finally, the tube was removed by the patient herself with a slight touch of the tube. The otoscopic images are shown in Figure 7.

## 4. Discussion

The parameter fR of the Medwave-PLAI test distinguishes ears with type A vs. type B tympanometry results. All other comparisons were not statistically different between types of tympanometry results, nor for parameter fR [Hz], nor for parameter P [10^−2^ mmho]. Due to the low statistical power, partly due to low group sample sizes and differences between group sizes, it is possible that a type II error is the main reason for not being able to differentiate between tympanogram types. Based on this finding, a differentiation between normal and non-movable eardrums seems possible. This offers the option to use the Medwave device for detecting liquid or infection (with or without effusion) in the middle ear, or a perforation of the eardrum (with or without tube). Additionally, it needs to be noted that type B includes many different medical causes. Therefore, in further investigations, a differentiation within type B should be considered. As only a few data points for groups of tympanometry types As, Ad, and C+ are given by this study, a reliable distinction among these groups is not feasible. As for clinical use, this is important; it should be evaluated in a future study. The shift in fR is in line with the resonance frequency in tympanometry changing in different ages of children [26]. A possible issue why P has no parameter to differentiate in tympanometry types may be because this value was subjectively observed to be more variable in repeated measurements, compared to fR being subjectively constant. The curve’s shape might be another value to consider in addition to fR. The curve shape may be an indicator for the reliability of the measurement, as a high P and a “clear” fR given by a clear maximum was seen in our experience in routine testing, while noisy results were shown in challenging scenarios like awake and moving toddlers.

The correlations between the patients’ age and both the parameter fR as well as the parameter P for the group of tympanometry result type A are in alignment with the offered age-related normative values for Medwave’s PLAI test. Whether the manufacturer’s currently used age-related normative values need to be optimized cannot be evaluated by our study’s results and therefore needs to be investigated in further studies, as well as considering such grouping also for different-aged patients when using tympanometry. In general, this approach to get normal values for multiple groups of different ages is not only interesting to re-evaluate in PLAI testing but could also be of interest to investigate in tympanometry, where only children under one year of age get an (additional) tympanometry using a 1000 Hz probe tone [22].

We did not experience any problems using the Medwave device for measuring, nor in exporting the reports to our clinical assistance sub via flash drive as a PDF file. Especially, the testing time was very short compared to tympanometry, which eases measurements in children. In addition, as Medwave solely uses an acoustic stimulation without any pressure, like in tympanometry, it avoids possible pain in patients or could even enable a measurement where tympanometry is not possible, like in patients shortly after certain ear surgeries. The statement regarding avoiding pain is based on the technical aspect that pressure in tympanometry might cause pain, and there is not one single report of pain in our data.

The case of the patient with paracentesis and later on paracentesis again plus insertion of a tube, highlights the possibilities of Medwave’s test results in the parameter fR and its similarity to the visual results from otoscopy and the measurement results from clinical routine measurement (the tympanometry). The results over time highlight the possibilities Medwave may offer in such or similar cases of patients. Further studies using a prospective approach should be performed to verify this illustrative case’s results, supported by the group comparison showing significant differences between tympanometry type A vs. tympanometry type B.

A measurement using the PLAI technique is possible when tympanometry is challenging to perform or when a measurement is not possible at all. This is especially helpful in children. It reduces or even avoids pain theoretically, as it does not need a sealed ear canal for PLAI, where sealing is obligatory in tympanometry. In contrast to these positive aspects, departments of otolaryngology must keep in mind that PLAI is not a replacement for tympanometry. Therefore, they will face additional costs for hardware and device maintenance. In contrast to research, this is an important point for clinical purposes. Further research will show whether PLAI can replace tympanometry or if additional functionality can be added to PLAI devices to make its purchase more profitable in clinical practice.

From young children, as shown in the introduction, tympanometry needs adaptation regarding the resonance frequency. In contrast, the daily routine in clinics does not differentiate in the tympanometry setup in adults, while Medwave PLAI does. With the type of tympanometry results in this study, we could analyze whether tympanometry is age-related in adults. This would be an interesting point for a future study.

The main limitation of the study is the small groups for tympanometry types As, Ad, and C+. Further studies are needed to investigate the tympanometry types in depth using a prospective approach, as this retrospective study showed how rare these results are, especially in comparison to types A and B. The hypothesis that Medwave is less painful than tympanometry needs to be evaluated in another prospective study due to a risk of bias in this retrospective data, as we had to rely on retrospective comments data, besides the technical aspect of no pressure when performing the Medwave measurement, where tympanometry needs pressure. This study was performed using an anonymous retrospective and monocentric approach. Due to this anonymous approach, possible data errors could not be rechecked. Due to the retrospective design, the study’s results are more susceptible to bias and confounding variables in comparison to a prospective study’s results. By its monocentric approach, the study could be biased by a few people performing the measurements and documentation in comparison to using a multicentric study design. Another pro for a prospective design in the future would be that re-verification of measurements would be possible. This way, it would also be possible to provide reliable performance metrics data, including sensitivity and specificity values. This future study should be double-blinded to exclude any kind of influence by patient bias or test person bias during the acquisition and interpretation of measurements. In such a prospective study, tone audiometry of ABR should be used to assess air and bone conduction values that could be correlated to the results of tympanometry and Medwave PLAI. In addition, future research could analyze inter-tester reliability in a prospective study design to address how the Medwave and tympanometry results might be influenced by the tester.

## 5. Conclusions

This study showed that using the Medwave device to perform a pressureless impedance measurement allows differentiation between tympanometry type A and tympanometry type B in clinical patients using the parameter fR (resonance frequency of the acoustic response recorded by the device’s microphone). Whether a differentiation between other tympanogram types may also be possible could not be finally found out due to the much smaller groups of the tympanogram types. This is due to the retrospective design and needs future evaluation. Type Ad and the group with sealing problems in tympanogram seem to have the highest probability for a possible differentiation to type A based on our limited data in the groups Ad and sealing problems. Age influences the parameters fR as well as P in group A. Influences of age in other groups were not found and need re-evaluation in future prospective studies. In summary, testing with the Medwave device shows promising results and was easy, quick, and more often performable in patients compared to tympanometry, being the clinical gold standard in middle ear analysis. Further measurements based on this pressureless technique, like stapedial reflex testing, may be interesting to investigate in the future. In summary, PLAI using Medwave can be used for testing the tympanic mobility without pressure and is not designed to replace tympanometry, but may be helpful where tympanometry is not possible.

## Figures and Tables

**Table 1:** Tympanometry types, their definition, and possible causes for each type [21,22,23].

Tympanometry Type	Definition	Possible Cause Resp. Causes
A	The central peak is of normal amplitude	None, eardrum vibrates normally
As	The central peak is of too low amplitude	Eardrum barely vibrates: Otosclerosis, thick tympanic membrane, or cholesteatoma?
Ad	The central peak is of too high amplitude	Eardrum vibrates too strongly: Ossicles separated, flaccid eardrum
B	No peak	Eardrum does not vibrate: Fluid or infection in the middle ear, middle ear infection with effusion, perforation of the eardrum, or ear tubes (depending on the volume analysis)?
C−	Shifted peak to the left (negative pressure)	Retracted eardrum: Tube ventilation disorder?
C+	Shifted peak to the right (positive pressure)	Protruding eardrum: Acute middle ear infection?

## Data Availability

The original contributions presented in this study are included in the article. Further inquiries can be directed to the corresponding author.

## Abbreviations

The following abbreviations are used in this manuscript:

fR	Value of the frequency at which the maximum value (P) of the admittance curve (module) occurs
MEMS	Micro–electro-mechanical systems
p	Pressure
P	Peak admittance value (module)
PLAI	Pressure Less Acoustic Immittance
v	Velocity
Ve	Equivalent volume of the ear canal
Y	Complex acoustic admittance
Z	Complex acoustic impedance
